# VEGF-A165b levels are reduced in breast cancer patients at primary diagnosis but increase after completion of cancer treatment

**DOI:** 10.1038/s41598-020-59823-5

**Published:** 2020-02-27

**Authors:** Maria Margarete Karsten, Maximilian Heinz Beck, Angela Rademacher, Julia Knabl, Jens-Uwe Blohmer, Julia Jückstock, Julia Caroline Radosa, Paul Jank, Brigitte Rack, Wolfgang Janni

**Affiliations:** 1 Department of Gynecology and Breast Care Center, University Hospital, Charité Universitätsmedizin Berlin, Berlin, Germany; 2 Department of Obstetrics and Gynecology, University Hospital, LMU Munich, Munich, Germany; 3 Department of Orthopedics, Schön Clinic, Munich, Harlaching Germany; 4 Department of Gynecology and Obstetrics, Saarland University Hospital, Homburg, Germany; 5 Department of Pathology, Philipps-University Marburg, Marburg, Germany; 6 Department of Gynecology and Obstetrics, University Hospital Ulm, Ulm, Germany

**Keywords:** Predictive markers, Prognostic markers, Breast cancer

## Abstract

The antiangiogenic splice variant VEGF-A165b is downregulated in a variety of cancer entities, but little is known so far about circulating plasma levels. The present analysis addresses this question and examines circulating VEGF-A/VEGF-A165b levels in a collective of female high-risk breast cancer patients over the course of treatment. Within the SUCCES-A trial 205 patients were recruited after having received primary breast surgery. Using ELISA VEGF-A/VEGF-A165b concentrations were determined and correlated to clinical characteristics (1) before adjuvant chemotherapy, (2) four weeks and (3) two years after therapy and compared to healthy controls (n = 107). VEGF_165b_ levels were significantly elevated after completion of chemotherapy. Within the breast cancer cohort, VEGF-A165b levels increased two years after completion of chemotherapy. VEGF-A plasma concentrations were significantly elevated in the breast cancer cohort at all examined time points and decreased after treatment. VEGF-A levels two years after chemotherapy correlated with increased cancer related mortality, no such correlation could be found between VEGF-A165b and the examined clinical characteristics. Compared to controls, VEGF-A/VEGF-A165b ratios were decreased in patients before and after chemotherapy. Our data suggests that circulating VEGF-A165b is significantly reduced in women with primary breast cancer at time of diagnosis; furthermore, levels change during adjuvant treatment.

## Introduction

Already in the 19th century, the great pathologist Rudolf Virchow described the abundant vascularization found in many tumors^1^. However, almost another century needed to pass until Senger discovered in 1983 a protein which led to a strong increase in the permeability of abdominal vasculature which he therefore named “*vascular permeability factor”*^2^. In 1989 Ferrara and colleagues found a heparin binding growth factor that specifically activated vascular endothelial cells and coined the term vascular endothelial growth factor VEGF^3^. Now we know that VEGF is not only one protein, but rather a family of growth factors with a similar protein structure but different abilities. Seven different human glycoproteins termed VEGF-A, VEGF-B, VEGF-C, VEGF-D, and placental like growth factor (PlGF) have been identified so far^4^.

VEGF-A is a family of different isoforms (VEGF-A121, VEGF-A165, VEGF-A183, VEGF-A189, VEGF-A206 and VEGF-A165b) with different functions and sizes. The coding gen for it lies on chromosome 6p21.3^5^ and consists of eight exons and seven introns^6^. Alternative splicing at exon six and seven as well as at the proximal end of exon 8a leads to a variety of proangiogenic isoforms of VEGF.

In 2002 Bates *et al*.^7^ were the first to describe a new splice variant that differed from the known ones in its biologic function. This splice variant named VEGF-A165b deviates from VEGF-A in six amino acids at the carboxy terminal 3′ end changing the exonic structure from CDKPRR to SLTRKD and from an angiogenic to antiangiogenic function^8^. It was postulated that the switch from a positive charged arginine in the proangiogenic isoform to the neutral aspartic acid and lysine in the antiangiogenic splice variant is the reason for a difference in function and altered binding to VEGF receptor 2^9^. In part, this seems to be due to a missing disulfide bridge because of the changed amino acid sequence and therefore the inability to bind the co-receptor Neuropilin-1, causing only a partial activation of the intracellular signaling cascade^10^.

There has been new work by Ganta *et al*. showing that VEGF-A165b also inhibits VEGF Receptor 1 related Signal Transduction and Activator of Transcription 3 (STAT3) signaling. VEGF-A165b is thereby able to decrease VEGF-A mediated activation of VEGFR1 causing a reduced neovascularization^11^. This is also of special interest as STAT3 and the IL-6/JAK2/STAT3 pathways have been identified as the key driver of CD44 + CD24 – cells that have stem cell like characteristics and are thought to be influential in metastasis and therapy resistance in advanced breast cancer^12–14^.

In the last twenty years, VEGF receptors have been identified as a key target in modern cancer therapies. Multiple substances aim at different domains of the receptor often by binding to the Adenosine Triphosphate (ATP) pocket of the protein kinase domain^15^. Antiangiogenic therapy therefore is a mainstay in treating multiple cancer entities. Substances like Bevacizumab as well as Tyrosine kinase inhibitors were approved for the treatment of various malignancies^16^. However antitumor response to Bevacizumab for breast cancer patients was not as hoped for, which lead to a revision regarding the Federal Drug Administrations (FDA) decision due to inconclusive results regarding the overall survival benefits^17^. These mixed results might be caused by a wrong patient selection because of a lack of markers to identify those breast cancer patients who might truly benefit from antiangiogenic therapy. There has been different studies looking at the role of VEGF splice variants in colon cancer and there relevance about resistance to Bevacizumab^18,19^.

While VEGF-A and its role in breast cancer has been studied extensively, not much is known regarding the splice variant VEGF-A165b in this cancer entity. To our knowledge, this is the first work that investigates VEGF-A165b levels in breast cancer patients before, during and after adjuvant chemotherapy.

## Methods

The aim of the present study was to further investigate the role of the vascular growth factors VEGF-A and VEGF-A165b as well as their distribution and possible prognostic value in patients with primary high-risk breast cancer. Therefore, we analyzed circulating blood levels of VEGF-A and VEGF-A165b in breast cancer patients after having received primary surgery (n = 205). Blood samples were collected before initiation and after completion of adjuvant chemotherapy and results were compared to healthy controls (n = 107).

### Study design

We recruited all our patients from the SUCCESS-A trial^20^ (EudraCT 2005-000490-21). The ethic committee of the Ludwig-Maximilians-University Munich approved the study. Written informed consent was obtained from all patients before study inclusion.

The SUCESS-A trial is a multi-center randomized Phase-III study that compares two different regimes of adjuvant chemotherapy for patients with high-risk breast cancer after having received either breast-conserving surgery or mastectomy. Between 2005 and 2007 3754 patients were included and randomized to two arms, receiving either three cycles of epirubicin, fluorouracil and cyclophosphamide followed by three cycles of docetaxel or three cycles of docetaxel combined with gemcitabine. After completion of chemotherapy, patients received endocrine therapy depending on the histological hormone receptor status of the tumor. Premenopausal patients were treated with tamoxifen for five years, while postmenopausal patients were treated with tamoxifen for two years followed by three years of anastrozol. HER2-positive patients received usually one year of trastuzumab. Further details are published elsewhere^21^.

### Patients

To achieve interindividual comparability we only analyzed patients, who received adjuvant chemotherapy with three cycles of epirubicin, fluorouracil and cyclophosphamide followed by docetaxel. Within the SUCCESS-A study 205 patients with available blood samples were recruited for our analysis. Patients had to meet the following inclusion and exclusion criteria, which correspond to those of the SUCCESS-A study:

### Inclusion criteria

Included were female patients with initial diagnosis of invasive epithelial breast cancer stage I-IIIC (pT1-4) according to the TNM classification system. Further inclusion criteria were the histological evidence of axillary lymph node metastasis or, for node-negative patients, one or more of the following high-risk criteria: age ≤ 35, pT ≥ 2, poor grading (G3) or negative hormone receptor status. R0-resection and good general medical condition for inclusion were also required. Minimum age for inclusion was at 18 years.

### Exclusion criteria

Patients with inflammatory breast cancer, distant metastasis (cM1) or secondary malignancies were excluded from this trial. Previous adjuvant, neoadjuvant cytotoxic, or radiotherapies were not permitted. Pregnant or breastfeeding women were also not included in this trial.

### Control group

We recruited a control cohort of 107 healthy premenopausal women between age 18 and 35. At the time of study inclusion, all women completed a questionnaire regarding their medical history. Women with pregnancy, recent surgery or major trauma within the last six months, acute infection within the last two months, long-term medications except of oral contraceptives, medical history for any kind of malignancy, or chronic inflammatory diseases were excluded from this study to avoid falsifying effects on VEGF plasma levels.

### Blood analysis

Peripheral venous blood samples were collected from breast cancer patients at predefined time points: (1) before the initiation of chemotherapy (blood sample one = BS 1), (2) within 28 days after completion of chemotherapy (blood sample two = BS 2) and (3) two years after chemotherapy (blood sample three = BS 3). Blood samples were collected once in the control cohort.

We compared VEGF-A and VEGF-A165b levels between breast cancer patients and controls. Furthermore, we analyzed changes in circulating VEGF-A and VEGF-A165b levels in the breast cancer cohort over the period of investigation. The measured VEGF-A and VEGF-A165b values were correlated with the clinical outcome parameters (cancer-related mortality and recurrence rate). The ratio of VEGF-A165b to total VEGF-A was calculated for patients with sufficient probe material ethylenediaminetetraacetic acid (EDTA) plasma) at all examined time points. VEGF-A165b/VEGF-A ratios of breast cancer patients were analyzed over the course of investigation and compared to those of controls.

As there is little data available regarding the appropriate probe material for circulating VEGF-A165b determination, blood levels of VEGF-A and VEGF-A165b were measured in EDTA and serum blood samples for both patients and controls. Using a matched samples analysis, we compared the used probe material (serum and EDTA-plasma) within the breast cancer and control cohort. All samples were measured in duplicate. Only samples with sufficient material for a duplicate analysis were included. Due to insufficient probe volume for a complete analysis or non-existence, probe numbers might vary between the examined time points.

### ELISA

Blood VEGF-A and VEGF-A165b levels were determined using a commercially available ultrasensitive sandwich ELISA kit (DuoSet Human VEGF165 (Cat. No. DY293B), DuoSet Human VEGF -A165b (Cat. No. DY3045); R&D Systems, Inc. (Minneapolis, USA)). In brief, monoclonal antibodies specific for the particular VEGF, coated onto a micro well plate, were used to sequester VEGF-A or VEGF-A165b from the added serum or EDTA samples. An enzyme-linked detection antibody specific for the particular VEGF was added, followed by a substrate solution for quantification. The optical density was determined immediately using a microplate reader (Dynatech-Laboratories (Guernsey, GB)). There is data showing that the above-mentioned Elisa might also detect the splice variant VEGF-A189b. However, the fraction probably is very small and there is currently no method to detect this specific splice^22^ variant and therefore it is not possible to determine the exact ratio. Detection limit for the VEGF-A165b Elisa was below 36 pg/ml.

### Statistical analysis

Statistics were calculated using R, Version 2.15.2 software. All determined VEGF-A and VEGF-A165b values are expressed as the arithmetic mean of the duplicate analysis. All data was examined for normal distribution. The Friedman test was applied on unconnected data samples and the Wilcoxon-Mann-Whitney tests on connected samples. A contingency table was calculated using the exact Fisher test and Chi-Square method. To illustrate the dependence between tumor characteristics and VEGF-A and VEGF-A165b distribution a multiple linear regression model was created. Survival curves were calculated according to the Kaplan-Meier method. Depending on VEGF-A and VEGF-A165b levels, patients were categorized into three groups at the respective time of blood collection:

#### Patients with low scores

≤ 25th quantile (Q0.25) for VEGF-A and 0 for VEGF-A165b.

#### Patients with intermediate scores

25th quantile to 75th quantile (Q0.25-Q0.75) for VEGF-A and > 75th Quantile (0-Q0.75) for VEGF-A165b.

#### Patients with high scores

>75th quantile (Q0.75) for VEGF-A and VEGF-A165b.

The significance level of all tests was defined to be alpha *<*0.05 (*<0.05; **<0.01; ***<0.001; ****<0.0001). The Holm-Bonferonni method was applied to correct for multiple comparison. Unless stated otherwise, all data is expressed as the mean ± SD. Detailed results are published in the supplementary section.

### Ethical approval

All applicable international, national and institutional ethics guidelines were followed. This article does not contain any studies with animals.

### Informed consent

Informed consent was obtained from all individual participants included in the study.

## Results

### Clinical characteristics

We included 205 patients with high-risk breast cancer after primary surgery and 107 healthy control subjects in this study. The observation period reached from the day of surgery to the time of death or the last observation date. The median follow-up time was 5.5 years. The average age at the first time of blood collection was 39 (21–65) years in the breast cancer cohort compared to 24 (18–44) years in the control cohort. All individuals of the control cohort were premenopausal women and most of the breast cancer patients were premenopausal (180/205). All breast cancer patients received adjuvant chemotherapy with 5-fluorouracil, epirubicin and cyclophosphamide, followed by docetaxel. Patients after breast conserving therapy (n = 141) received mandatory radiotherapy, patients after mastectomy (n = 64) only in cases of coexisting risk factors. In total, 180 patients received adjuvant radiation starting after chemotherapy. Premenopausal patients with positive hormone receptor status received endocrine therapy with tamoxifen for five years (n = 121), postmenopausal patients tamoxifen for two years, followed by anastrozole for further three years (n = 22). Patients with positive human epidermal growth factor receptor 2 (HER2) receptor status received trastuzumab for one year (n = 55). Further details are shown in Table 1.

**Table 1:** Clinical-pathological characteristics.

	n (%)		n (%)
Axillary lymph node involvement		Histology^†^	
nodal-negative (N0)	87 (42)	carcinoma of no specific type	180 (88)
1–3 involved lymph nodes	84 (41)	invasive lobular carcinoma	12 (6)
4–9 involved lymph nodes	26 (13)	other carcinoma	12 (6)
≥ 10 involved lymph nodes	8 (4)	Grading^†^	
Hormone receptor status		G1	5 (2)
Negative	62 (30)	G2	95 (46)
Positive	143 (70)	G3	104 (52)
Estrogen receptor statusa		HER2 status^†^	
negative	74 (36)	negative	139 (68)
positive	130 (64)	positive	62 (32)
Progesterone receptor status^a^		Recurrence free survival	
Negative	85 (41)	no recurrence	168 (82)
Positive	119 (59)	recurrence	37 (18)
Tumor size^a^		Overall survival	
pT1a-c	95 (46)	survived	186 (91)
pT2	100 (49)	deceased	19 (9)
pT3	8 (5)		

^a^Hormone receptor status, histology and grading not known in one case. Tumor size not known in two cases. HER2 status not known in four cases.

### Probe material associated differences between serum and EDTA plasma levels

**Figure 1:**
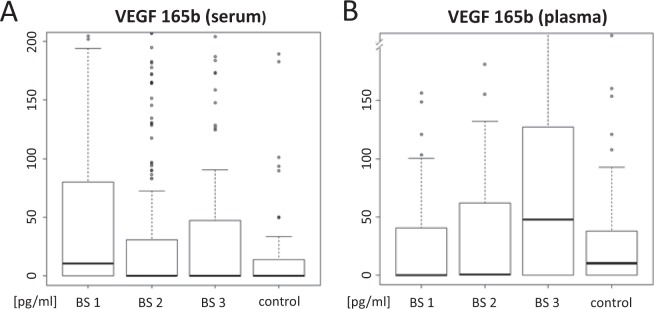
Probe associated material differences. (**A**) Patients’ VEGF-165b serum levels are plotted in a box plot diagram over the course of treatment: initiation of chemotherapy (blood sample 1 = BS 1), after completion of chemotherapy (blood sample 2 = BS 2) and two years after completion of therapy (blood sample 3 = BS 3) and for healthy controls (control). (**B**) Accordingly, VEGF-165b plasma values are displayed.

At all examined time points, VEGF-A serum levels have been found to be significantly higher than EDTA plasma values, both for breast cancer patients (p < 0.0001) and healthy controls (p < 0.0001) as can be seen in Fig. 1. Similar results have been published before^27^. Within the control cohort VEGF-A165b EDTA plasma levels were elevated compared to serum levels (p < 0.0001). Results for the breast cancer cohort are more heterogeneous: Before the initiation of chemotherapy VEGF-A165b, serum levels were significantly higher than plasma concentrations (p < 0.0001). The results of the second and third blood collection were more in line with observations in the control group. Blood samples after completion of chemotherapy tended to be higher in EDTA plasma, but this difference did not reach statistical significance (p = 0.195). Two years after completion of chemotherapy, VEGF-A165b levels in EDTA plasma were again significantly higher than serum levels (p < 0.0001).

We focused our further analysis on VEGF plasma levels to minimize falsifying effects caused by thrombocyte VEGF production^28^. Only EDTA plasma levels are reported in the further section. Results for VEGF-A and VEGF-A165b serum levels are shown in the supplementary section.

#### VEGF-A

We found a broad interindividual variability of VEGF-A plasma levels, both for breast cancer patients and healthy controls. At all studied time points VEGF-A plasma concentrations were significantly elevated in the breast cancer cohort compared to the control group [p (BS 1/2/3) < 0.0001]. Furthermore, breast cancer patients showed significantly less non-measurable values (0) at all times of blood collection (p (BS 1/2/3) < 0.0001].

**Figure 2:**
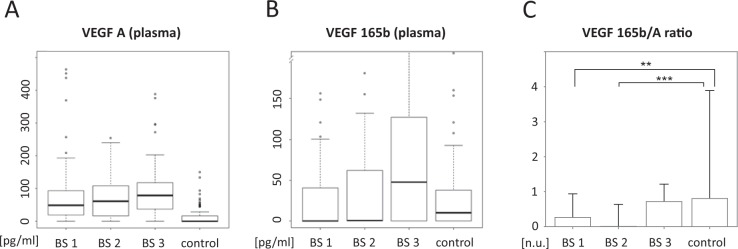
VEGF-A and VEGF-165b plasma levels. (**A**) Box plot diagram of VEGF-A plasma levels for breast cancer patients before initiation of chemotherapy (blood sample 1 = BS 1), after completion of chemotherapy (blood sample 2 = BS 2), two years after completion of therapy (blood sample 3 = BS 3) and for healthy controls (control). (**B**) Accordingly, VEGF-165b plasma values are displayed. (**C**) Ratio of VEGF-165b to VEGF-A plasma levels for breast cancer patients over the course of treatment and for healthy controls. Data is displayed as median and 95% Confidence interval (n.u. = no unit, **p < 0.01, ***p < 0.001).

Within the breast cancer cohort, we found no significant difference in VEGF-A concentrations before and after chemotherapy as shown in Fig. 2. Two years after completion of chemotherapy, VEGF-A plasma levels were significantly elevated compared to the blood collection before initiation of chemotherapy (p = 0.027). However, no significant difference was found between blood sample two and blood sample three (p = 1.000).

#### VEGF-165b

We found a broad interindividual variability of VEGF-A165b plasma levels, both for breast cancer patients and healthy controls.

VEGF-A165b concentrations after completion of chemotherapy tended to be significantly higher than those in the control cohort (p = 0.040). No detectable differences could be found between the other examined blood samples (BS 1 and BS 3) and controls. Interestingly, breast cancer patients showed significantly more often non-measurable values (0) at blood sample one and two compared to controls (p (BS 1) = 0.030, p (BS 2) < 0.001]. The number of patients with measurable values increased again at blood sample three two years after completion of chemotherapy and no significant difference could be detected anymore (p = 0.567).

Within the breast cancer cohort, VEGF-A165b plasma levels were significantly increased two years after completion of chemotherapy compared to the blood samples before chemotherapy (p < 0.0001). There was no significant difference between blood sample one and two (p = 0.330).

### Correlation between clinical characteristics and VEGF-A/VEGF-A165b_b_ plasma levels

As described in the method section, breast cancer patients were categorized into three groups depending on their VEGF-A and VEGF-A165b plasma values: Patients with low, intermediate or high plasma levels.

**Figure 3:**
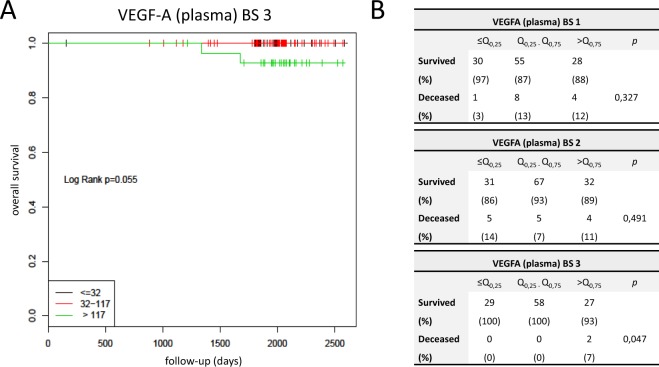
Overall survival in dependency of VEGF-A. (**A**) Showing a Kaplan-Meyer plot: The overall survival is shown in dependency of the VEGF-A plasma levels two years after completion of chemotherapy. Low VEGF-A values are plotted in black, intermediate in red and high values in green. (**B**) The tables are showing detailed data about overall survival in relationship to the VEGF-A levels before initiation of chemotherapy (blood sample 1 = BS 1), after completion of chemotherapy (blood sample 2 = BS 2) and two years after completion of therapy (blood sample 3 = BS 3).

We could not detect any dependency between VEGF-A levels and relapse rate at any of the examined time points (p > 0.05, Log Rank test). While high VEGF-A levels did not correlate with cancer related mortality rate at the blood samples collected before and after chemotherapy (blood sample one and two), a significant correlation between high plasma VEGF-A levels and increased cancer related mortality rate was found at blood sample three (two years after completion of chemotherapy), Fig. 3. It is noteworthy that significance was reached by only two deaths in the high plasma level group (7% mortality rate, 2/29) compared to no deaths in the low (0/29) and intermediate level group (0/58).

No relationship was found between VEGF-A165b values and relapse-rate or overall survival at any of the examined time points (p > 0.05, Log Rank test).

### VEGF-A165b***/VEGF-A ratios***

Compared with healthy controls, the ratio of plasma VEGF-A165b to VEGF-A was significantly lower in breast cancer patients before the initiation of chemotherapy (p = 0.0026 (BS1)) and at the blood sample after completion of chemotherapy (p = 0.0004 (BS2). No significant differences were detected between controls and study patients two years after completion of chemotherapy.

Within the breast cancer cohort, VEGF-A165b/VEGF-A ratios did not significantly differ before and after chemotherapy. Notably a statistical trend could be observed in the matched paired analysis (p = 0.0827).

## Discussion

We found VEGF-A levels to be elevated at all examined time points in the breast cancer cohort compared to healthy individuals, these results are in line with previously published data^23^. Therefore, we focus our further interest on VEGF-A165b lacking so far on broad clinical data.

VEGF-A165b was first described by Bates *et al*.^7^ in 2002 and was discovered in renal tissue noting that there was a PCR band less often found in tumor tissue than in the surrounding healthy tissue^7^. Since then, it has been described in a variety of tissues and shown to be downregulated in cancer^8^ and diabetic retinopathy^24^ but it is upregulated in conditions like systemic sclerosis and peripheral artery disease (PAD)^11^. VEGF-A165b is described, as the dominant splice variant in healthy tissue but there seems to be a switch to the pro-angiogenic version in the case of tumor development^7,25,26^. Furthermore, many epithelial tumors show reduced levels of VEGF-A165b compared to healthy tissue^10^. Most work in trying to understand the role and function of VEGF-A165b has been done in tissue or animal models and only three studies so far have investigated circulating VEGF-A165b levels in humans, one in the blood of healthy individuals^8^, one in women with preeclampsia^27^ and one in patients with colon cancer^19^.

To our knowledge, we are the first to show VEGF-A165b levels in the blood of breast cancer patients before, during, and after their cancer treatment. Besides matching VEGF-A165b levels to clinical tumor characteristics, we compared them to blood levels found in a cohort of healthy women.

There was a wide distribution of VEGF-A165b levels in both groups. Out of 95 healthy women, 34% (32/95) had undetectable levels. This finding is in line with previous published data where as much as 48% (11/23) patients had undetectable blood levels^8^. This is surprising since VEGF-A165b was described as the dominant splice variant in tissue^28^. Concordantly, one could expect detectable plasma levels in all examined probes, a possible explanation for the discrepancy between tissue expression and circulating levels could be that VEGF-A165b acts more as a local mediator than as a soluble and circulating protein. Another or additional explanation might be an insufficient sensitivity of the available ELISA Kit for which the detection limit in our experiments was 36 pg/ml. On the other side, we also found very high VEGF-A165b levels in selected individuals, reaching up to 1354 pg/ml. Despite the broad range of VEGF-A165b levels, we are able to demonstrate a significant difference between breast cancer patients and healthy controls.

Breast cancer patients showed significantly more often an undetectableVEGF-A165b level before and after completion of chemotherapy compared to healthy women. Furthermore, at time point three two years after primary surgery the levels of VEGF-A165b has risen significantly compared to the first two time points, while there was no significant difference compared to the control group. Interestingly, the 75% Quantile of the levels measured in the breast cancer cohort was at time point three above the levels found at the healthy cohort.

Bunni *et al*. showed in colon cancer patients that the ratio of VEGF-Axxxb to pan VEGF could be useful as a biomarker to select those patients, who might benefit from Bevacizumab therapy. They were able to show a correlation between progression free survival and less than median VEGF-Axxxb to pan VEGF ratio^19^. While we couldn’t demonstrate a significant difference regarding the VEGF-A165b to VEGF-A ratio, we found a statistically highly significant difference between breast cancer patients and control group. The lacking statistical difference between the patient’s groups might be explained by patient selection and comparatively few recurrences and deaths. Notably, there was a statistical trend for a change of the VEGF-A165b to VEGF-A ratio during and after treatment. One questions that needs further work and validation in a larger cohort is to determine what the “average” VEGF-A165b to VEGF-A level for breast cancer patients is and if this correlates with outcome following Bevacizumab treatment.

The findings of this study demonstrate, despite a wide inter individual range of VEGF-A165b levels, statistically significant differences between healthy individuals and breast cancer patients and a change of VEGF-A165b levels over the course of cancer treatment to reach normal or above normal levels two years after finishing treatment. If those initially reduced VEGF-A165b levels are caused by breast cancer or are induced by surgery and chemotherapy cannot be concluded at this point. Bunni *et al*. described no significant changes in VEGF-A levels before and after surgery in a collective of 18 patients with colon cancer. So, one might speculate that the observed reduction of VEGF-A165b might be induced by tumor related mechanisms Further studies with larger patient collectives should address this question. Therefore, it will be important to examine VEGF-A165b levels at primary diagnosis of breast cancer before any medical or surgical intervention. However, the fact that a large percentage of women in the breast cancer cohort had at time point one undetectable levels is in concordance with the fact that the expression of VEGF-A165b is downregulated in a large variety of cancers^8,10,29,30^. A possible explanation might be that the protein is downregulated at tissue level and thereby reduced in the soluble compartment to a level where it is undetectable by the available ELISA kit.

The identification of predictive markers concerning antiangiogenic cancer therapies is an important goal. Bates *et al*. were already able to show that a low VEGF-A165b /VEGF ratio was a predictor for a better response to Bevacizumab in metastatic colon cancer patients^31^. If VEGF-A165b could be, a useful surrogate marker to select patients for antiangiogenic therapies needs still to be evaluated in further studies and examined in a randomized controlled trial.

## Supplementary information

Supplementary information.
