# Supplementary material for: VEGF-A165b levels are reduced in breast cancer patients at primary diagnosis but increase after completion of cancer treatment
Source: Sci Rep. 2020 Feb 27;10:3635. doi: 10.1038/s41598-020-59823-5 (PMC7046696; doi:10.1038/s41598-020-59823-5)
Supplement: Supplementary file 1 — Supplementary information. [file 41598_2020_59823_MOESM1_ESM.docx]

**Supplementary section for SREP-19-30669A**

**VEGF-A165b levels are reduced in breast cancer patients at primary diagnosis but increase after completion of cancer treatment**

Karsten Maria Margarete^1^ *, Beck Maximilian Heinz^1^ *, Rademacher Angela^2/3^, Knabl Julia^2^, Blohmer Jens-Uwe^1^, Jückstock Julia^2^, Radosa Julia Caroline^4^, Jank Paul^5^, Rack Brigitte ^2/6^, Janni Wolfgang^6^

**Affiliations**

1 Department of Gynecology and Breast Care Center, University Hospital, Charité Universitätsmedizin Berlin, Berlin, Germany

2 Department of Obstetrics and Gynecology, University Hospital, LMU Munich, Munich, Germany

3 Department of Orthopedics, Schön Clinic, Munich Harlaching, Germany

4 Department of Gynecology and Obstetrics, Saarland University Hospital, Homburg, Germany

5 Department of Pathology, Philipps-University Marburg, Marburg, Germany

6 Department of Gynecology and Obstetrics, University Hospital Ulm, Ulm, Germany

**Corresponding author**

Maria Margarete Karsten

Klinik für Gynäkologie mit Brustzentrum

Charité Universitätsmedizin Berlin

Charitéplatz 1

10117 Berlin

+ 49 30 450 664 279

[Maria-margarete.karsten@charite.de](mailto:Maria-margarete.karsten@charite.de)

* Maria Margarete Karsten and Maximilian Heinz Beck contributed equally to the manuscript

***VEGF-A serum results***

In the following section only results for VEGF-A_/165b_ serum values are reported.

***VEGF-A***

At all studied time points VEGF-A serum levels were significantly elevated in the breast cancer cohort compared to the control group [p (BS 1/2/3) <0.0001]. Furthermore, breast cancer patients showed significantly less non-measurable values ​​(0) at all times of blood collection (p(BS 1/2)<0,0001, p(BS 3)=0,006)

Within the breast cancer cohort, VEGF-A serum concentrations after completion of chemotherapy at blood sample two were significantly elevated compared to levels before chemotherapy (BS1, p=0,002) and also to levels two years after completion of chemotherapy (BS 3, p=0.002). However, no significant difference was found between blood sample one and blood sample three (p=0.319).

***VEGF-A165b***

We found a broad interindividual variability of VEGF-A165b serum levels, both for breast cancer patients and healthy controls.

Serum VEGF-A165b concentrations before the initiation of chemotherapy (BS 1) tended to be significantly higher than those in the control cohort (p<0,0001). No detectable differences could be found between the examined blood samples after completion of chemotherapy (BS 2 and BS 3) and controls.

Within the breast cancer cohort, VEGF-A165b serum levels before the initiation of chemotherapy (BS 1) were also significantly increased compared to blood samples after completion of chemotherapy (BS 2, p<0.0001), but not compared to levels two years later (BS 3, p=0,286). There were no significant differences between the examined blood samples after completion of chemotherapy (BS 2 and BS 3, p=0,286).

***Correlation between clinical characteristics and VEGF-A/A165 serum levels***

We could not detect any dependency between VEGF-A serum levels and recurrence rate at any of the examined time points (p>0,05, Log Rank test). While high VEGF-A levels did not correlate with cancer related mortality rate at the blood samples collected before and after chemotherapy (blood sample one and two), a significant correlation between high serum VEGF-A levels and increased cancer related mortality rate was found at blood sample three (two years after completion of chemotherapy). It is noteworthy that significance was reached by only two deaths in the high serum level group (6 % mortality rate, 2/31) compared to no deaths in the low (0/32) and intermediate level group (0/62).

No relationship was found between VEGF-A165b serum values and recurrence-rate or overall survival at any of the examined time points (p>0,05, Log Rank test).

**Detailed results**

***VEGF-A***

Supplementary Table 1. VEGF-A in breast cancer patients.

| **VEGF-A** | **(pg/ml)** | **Min.** | **(Q_0,25_)** | **(Q_0,50_)** | **Mean ± SD** | **(Q_0,75_)** | **(Q_0,95_)** | **Max.** |
| --- | --- | --- | --- | --- | --- | --- | --- | --- |
| **Serum** | BS 1 (n=180) | 0 | 98,6 | 156,9 | 182,5 ± 9,5 | 238,4 | 416,8 | 907,9 |
|  | BS 2 (n=166) | 0 | 98,1 | 181,7 | 213,9 ± 14,0 | 278,2 | 513,2 | 1640 |
|  | BS 3 (n=125) | 0 | 74,2 | 143,2 | 168,8 ± 13,0 | 212,2 | 401,8 | 1044 |
| **Plasma** | BS 1 (n=127) | 0 | 17,8 | 54,0 | 82,9 ± 8,3 | 101,8 | 267,0 | 463,8 |
|  | BS 2 (n=145) | 0 | 14,5 | 60,9 | 81,0 ± 8,5 | 112,6 | 237,9 | 803,9 |
|  | BS 3 (n=116) | 0 | 31,6 | 70,7 | 95,3 ± 9,8 | 117,2 | 298,1 | 654,4 |

(number [n]; Blood sample 1/2/3 [BS 1/2/3]; minimal value [Min.]; standard deviation [MW ± SD]; maximal value [Max.]; quantile [Q]; median [ Q_0,50_]) All values in pg/ml.

Supplementary Table 2. VEGF-A in healthy individuals.

| **VEGF-A** | **(pg/ml)** | **Min.** | **(Q_0,25_)** | **(Q_0,50_)** | **Mean ± SD** | **(Q_0,75_)** | **(Q_0,95_)** | **Max.** |
| --- | --- | --- | --- | --- | --- | --- | --- | --- |
| **Serum** | (n=97) | 0 | 23,3 | 66,2 | 94,1 ± 15,9 | 124,5 | 222,2 | 1479 |
| **Plasma** | (n=93) | 0 | 0 | 0 | 37,3 ± 17,5 | 16,3 | 86,9 | 1497 |

(number [n]; minimal value [Min.]; standard deviation [MW ± SD]; maximal value [Max.]; quantile [Q]; median [ Q_0,50_]) All values in pg/ml.

## *VEGF-A165b*

Supplementary Table 3. VEGF-A165b in breast cancer patients.

| **VEGF-A165b** | **(pg/ml)** | **Min.** | **(Q_0,25_)** | **(Q_0,50_)** | **Mean ± SD** | **(Q_0,75_)** | **(Q_0,95_)** | **Max.** |
| --- | --- | --- | --- | --- | --- | --- | --- | --- |
| **Serum** | BS 1 (n=178) | 0 | 0 | 10,5 | 68,2 ± 10,1 | 79,6 | 302,4 | 969,7 |
|  | BS 2 (n=155) | 0 | 0 | 0 | 36,0 ± 6,2 | 30,6 | 178,8 | 601,8 |
|  | BS 3 (n=122) | 0 | 0 | 0 | 55,1 ± 13,2 | 47,3 | 266,8 | 1188 |
| **Plasma** | BS 1 (n=102) | 0 | 0 | 0 | 41,2 ± 8,1 | 48,7 | 156,7 | 510,2 |
|  | BS 2 (n=137) | 0 | 0 | 0 | 34,1 ± 6,5 | 44,9 | 163,5 | 657,2 |
|  | BS 3 (n=118) | 0 | 0 | 19,5 | 97,8 ± 21,0 | 120,1 | 303,4 | 1947 |

(number [n]; Blood sample 1/2/3 [BS 1/2/3]; minimal value [Min.]; standard deviation [MW ± SD]; maximal value [Max.]; quantile [Q]; median [ Q_0,50_]) All values in pg/ml.

Supplementary Table 4. VEGF-A165b in healthy individuals.

| **VEGF-A** | **(pg/ml)** | **Min.** | **(Q_0,25_)** | **(Q_0,50_)** | **Mean ± SD** | **(Q_0,75_)** | **(Q_0,95_)** | **Max.** |
| --- | --- | --- | --- | --- | --- | --- | --- | --- |
| **Serum** | (n=100) | 0 | 0 | 0 | 53,1 ± 21,3 | 13,9 | 183,0 | 1439 |
| **Plasma** | (n=95) | 0 | 0 | 10,1 | 66,4 ± 21,7 | 37,6 | 173,8 | 1354 |

(number [n]; minimal value [Min.]; standard deviation [MW ± SD]; maximal value [Max.]; quantile [Q]; median [ Q_0,50_]) All values in pg/ml.

Supplementary Table 5. Plasma VEGF-A165b/A ratios

| **VEGF-A** | **(pg/ml)** | **Min.** | **(Q_0,25_)** | **(Q_0,50_)** | **Mean ± SD** | **(Q_0,75_)** | **(Q_0,95_)** | **Max.** |
| --- | --- | --- | --- | --- | --- | --- | --- | --- |
| **Healthy** | (n=33) | 0 | 0.4579 | 0.8181 | 5.983 ± 13.61 | 13,9 | 35.42 | 75,42 |
| **BS1** | (n=42) | 0 | 0 | 0.2724 | 1.486 ± 2.876 | 1.379 | 8.58 | 13.37 |
| **BS2** | (n=42) | 0 | 0 | 0.1762 | 4.072 ± 15.6 | 0.9766 | 35.49 | 94.17 |
| **BS3** | (n=42) | 0 | 0 | 0.7299 | 2.204 ± 14.41 | 1.837 | 14.41 | 25.72 |
|  |  |  |  |  |  |  |  |  |

(number [n]; minimal value [Min.]; standard deviation [MW ± SD]; maximal value [Max.]; quantile [Q]; median [ Q_0,50_]) All values in pg/ml.

***Contingency tables***

Supplementary Table 5. Serum VEGF-A in breast cancer patients.

|  | **VEGF-A (serum)** | | | | | | | | | | | | | | | | | |
| --- | --- | --- | --- | --- | --- | --- | --- | --- | --- | --- | --- | --- | --- | --- | --- | --- | --- | --- |
|  | n | | | Q_0,50_ (pg/ml) | | | mean (pg/ml) | | | SE | | | R | | | p-value | | |
| Blood sample | 1 | 2 | 3 | 1 | 2 | 3 | 1 | 2 | 3 | 1 | 2 | 3 | 1 | 2 | 3 | 1 | 2 | 3 |
| **Nodal status** |  | | | | | | | | | | | | | | | | | |
| nodal-negative (N0) | 80 | 69 | 58 | 171 | 191 | 152 | 195 | 211 | 174 | 15 | 17 | 16 |  |  |  |  |  |  |
| 1-3 involved lymph nodes | 70 | 67 | 48 | 156 | 177 | 126 | 173 | 229 | 163 | 14 | 29 | 26 | -17 | -15 | -28 | 0,489 | 0,678 | 0,428 |
| 4-9 involved lymph nodes | 18 | 18 | 12 | 152 | 170 | 152 | 160 | 185 | 177 | 27 | 31 | 42 | -61 | -64 | -7 | 0,104 | 0,249 | 0,891 |
| ≥ 10 involved lymph nodes | 8 | 7 | 5 | 152 | 186 | 167 | 213 | 228 | 189 | 58 | 49 | 36 | 9 | -15 | 13 | 0,855 | 0,839 | 0,860 |
| **Hormon receptor status** |  | | | | | | | | | | | | | | | | | |
| negative | 53 | 44 | 36 | 174 | 220 | 150 | 208 | 224 | 186 | 21 | 20 | 22 |  |  |  |  |  |  |
| positive | 123 | 117 | 87 | 154 | 177 | 146 | 173 | 214 | 164 | 11 | 18 | 16 | 39 | -28 | -153 | 0,513 | 0,751 | 0,075 |
| **Estrogen receptor status** |  | | | | | | | | | | | | | | |  | | |
| negative | 62 | 53 | 41 | 176 | 193 | 143 | 213 | 219 | 176 | 19 | 21 | 21 | 70 | -15 | -92 | 0,132 | 0,828 | 0,195 |
| positive | 114 | 108 | 82 | 152 | 179 | 146 | 168 | 215 | 167 | 10 | 19 | 17 |  |  |  |  |  |  |
| **Progesterone receptor status** |  | | | | | | | | | | | | | | | | | |
| negative | 72 | 59 | 51 | 165 | 213 | 157 | 194 | 223 | 174 | 17 | 20 | 17 | -15 | 3 | -92 | 0,647 | 0,955 | 0,195 |
| positive | 104 | 102 | 72 | 156 | 178 | 141 | 177 | 213 | 168 | 12 | 20 | 19 |  |  |  |  |  |  |
| **Tumorsize** |  | | | | | | | | | | | | | | | | | |
| pT1a-c | 86 | 73 | 59 | 157 | 173 | 127 | 171 | 197 | 152 | 12 | 17 | 17 |  |  |  |  |  |  |
| pT2 | 83 | 82 | 59 | 162 | 210 | 162 | 201 | 237 | 190 | 16 | 24 | 20 | 23 | 41 | 29 | 0,272 | 0,198 | 0,327 |
| pT3 | 7 | 6 | 5 | 99 | 160 | 104 | 142 | 171 | 161 | 33 | 37 | 71 | -25 | --24 | 62 | 0,652 | 0,781 | 0,408 |
| **Histology** |  | | | | | | | | | | | | | | | | | |
| Invasive ductal carcinoma | 155 | 140 | 108 | 164 | 186 | 143 | 188 | 218 | 171 | 10 | 16 | 14 |  |  |  |  |  |  |
| Invasive lobular carcinoma | 11 | 10 | 7 | 151 | 216 | 146 | 152 | 239 | 130 | 42 | 56 | 39 | -22 | 50 | -59 | 0,615 | 0,460 | 0,338 |
| **Grading** |  | | | | | | | | | | | | | | | | | |
| G1 | 5 | 4 | 3 | 154 | 133 | 59 | 120 | 121 | 139 | 39 | 15 | 83 |  |  |  |  |  |  |
| G2 | 78 | 76 | 59 | 151 | 185 | 147 | 182 | 237 | 187 | 15 | 27 | 23 | 42 | 66 | 39 | 0,500 | 0,500 | 0,675 |
| G3 | 93 | 81 | 61 | 163 | 186 | 143 | 189 | 202 | 156 | 14 | 12 | 14 | 33 | 22 | -17 | 0,603 | 0,822 | 0,856 |
| **HER2/neu status** |  | | | | | | | | | | | | | | | | | |
| negative | 122 | 109 | 80 | 172 | 181 | 142 | 189 | 205 | 169 | 12 | 14 | 15 | 10 | -40 | -20 | 0,645 | 0,230 | 0,514 |
| positive | 54 | 51 | 40 | 153 | 185 | 147 | 171 | 237 | 181 | 15 | 34 | 26 |  |  |  |  |  |  |

(number [n], median [Q_0,50_], standard error [SE], regression coefficient [R])

Supplementary Table 6. **Plasma VEGF-A in breast cancer patients.**

|  | **VEGF A** **(plasma)** | | | | | | | | | | | | | | | | | |
| --- | --- | --- | --- | --- | --- | --- | --- | --- | --- | --- | --- | --- | --- | --- | --- | --- | --- | --- |
|  | n | | | Q_0,50_ (pg/ml) | | | mean (pg/ml) | | | SE | | | R | | | p-value | | |
| Blood sample | 1 | 2 | 3 | 1 | 2 | 3 | 1 | 2 | 3 | 1 | 2 | 3 | 1 | 2 | 3 | 1 | 2 | 3 |
| **Nodal status** |  | | | | | | | | | | | | | | | | | |
| nodal-negative (N0) | 59 | 61 | 54 | 48 | 54 | 69 | 79 | 79 | 92 | 12 | 10 | 11 |  |  |  |  |  |  |
| 1-3 involved lymph nodes | 45 | 60 | 46 | 58 | 52 | 66 | 85 | 74 | 98 | 15 | 12 | 17 | -1 | -11 | 10 | 0,955 | 0,620 | 0,698 |
| 4-9 involved lymph nodes | 13 | 13 | 11 | 50 | 64 | 85 | 93 | 112 | 110 | 34 | 59 | 56 | 5 | 17 | 21 | 0,871 | 0,633 | 0,587 |
| ≥ 10 involved lymph nodes | 5 | 7 | 4 | 76 | 114 | 91 | 102 | 133 | 77 | 28 | 35 | 28 | 5 | 35 | 2 | 0,914 | 0,433 | 0,968 |
| **Hormon receptor status** |  | | | | | | | | | | | | | | | | | |
| negative | 41 | 40 | 34 | 68 | 68 | 106 | 87 | 88 | 113 | 15 | 13 | 15 |  |  |  |  |  |  |
| positive | 81 | 101 | 81 | 50 | 55 | 59 | 82 | 80 | 88 | 11 | 11 | 13 | 45 | 57 | -40 | 0,361 | 0,331 | 0,515 |
| **Estrogen receptor status** |  | | | | | | | | | | | | | | | | | |
| negative | 50 | 46 | 39 | 57 | 64 | 103 | 94 | 94 | 110 | 15 | 15 | 15 | 56 | 61 | -4 | 0,121 | 0,180 | 0,943 |
| positive | 72 | 95 | 76 | 54 | 55 | 61 | 77 | 77 | 88 | 10 | 11 | 13 |  |  |  |  |  |  |
| **Progesterone receptor status** |  | | | | | | | | | | | | | | | | | |
| negative | 52 | 52 | 47 | 64 | 65 | 80 | 81 | 86 | 106 | 12 | 11 | 13 | 12 | 7 | 1 | 0,737 | 0,826 | 0,969 |
| positive | 70 | 89 | 68 | 49 | 55 | 55 | 86 | 80 | 88 | 12 | 12 | 14 |  |  |  |  |  |  |
| **Tumorsize** |  | | | | | | | | | | | | | | | | | |
| pT1a-c | 61 | 67 | 60 | 48 | 45 | 59 | 71 | 72 | 185 | 11 | 11 | 11 |  |  |  |  |  |  |
| pT2 | 57 | 69 | 51 | 70 | 72 | 85 | 100 | 93 | 107 | 14 | 14 | 18 | 24 | 10 | 10 | 0,203 | 0,592 | 0,639 |
| pT3 | 4 | 5 | 4 | 51 | 79 | 58 | 49 | 74 | 101 | 12 | 16 | 57 | -24 | -24 | 38 | 0,231 | 0,665 | 0,535 |
| **Histology** |  | | | | | | | | | | | | | | | | | |
| Invasive ductal carcinoma | 105 | 121 | 101 | 54 | 61 | 71 | 84 | 82 | 96 | 10 | 10 | 11 |  |  |  |  |  |  |
| Invasive lobular carcinoma | 8 | 9 | 5 | 75 | 88 | 72 | 118 | 113 | 75 | 35 | 36 | 19 | 48 | 43 | -36 | 0,225 | 0,299 | 0,510 |
| **Grading** |  | | | | | | | | | | | | | | | | | |
| G1 | 4 | 4 | 3 | 8 | 34 | 46 | 19 | 40 | 48 | 13 | 13 | 4 |  |  |  |  |  |  |
| G2 | 56 | 65 | 56 | 69 | 61 | 71 | 101 | 91 | 109 | 15 | 17 | 18 | 69 | 27 | 31 | 0,196 | 0,639 | 0,637 |
| G3 | 62 | 72 | 56 | 51 | 65 | 74 | 72 | 77 | 85 | 10 | 8 | 9 | 24 | 4 | -1 | 0,660 | 0,950 | 0,994 |
| **HER2/neu status** |  | | | | | | | | | | | | | | | | | |
| negative | 84 | 96 | 75 | 59 | 54 | 67 | 90 | 82 | 97 | 13 | 11 | 12 | 22 | -11 | -9 | 0,275 | 0,591 | 0,684 |
| positive | 38 | 44 | 37 | 49 | 78 | 80 | 70 | 84 | 100 | 11 | 12 | 18 |  |  |  |  |  |  |

(number [n], median [Q_0,50_], standard error [SE], regression coefficient [R])

Supplementary Table 7. Serum VEGF-A165b in breast cancer patients.

|  | **VEGF-A165b (serum)** | | | | | | | | | | | | | | | | | |
| --- | --- | --- | --- | --- | --- | --- | --- | --- | --- | --- | --- | --- | --- | --- | --- | --- | --- | --- |
|  | n | | | Q_0,50_ (pg/ml) | | | mean (pg/ml) | | | SE | | | R | | | p-value | | |
| Blood sample | 1 | 2 | 3 | 1 | 2 | 3 | 1 | 2 | 3 | 1 | 2 | 3 | 1 | 2 | 3 | 1 | 2 | 3 |
| **Nodal status** |  | | | | | | | | | | | | | | | | | |
| nodal-negative (N0) | 80 | 60 | 59 | 10 | 0 | 0 | 66 | 32 | 63 | 14 | 9 | 18 |  |  |  |  |  |  |
| 1-3 involved lymph nodes | 68 | 65 | 45 | 9 | 0 | 0 | 65 | 41 | 56 | 17 | 12 | 27 | -11 | 15 | -12 | 0,675 | 0,359 | 0,737 |
| 4-9 involved lymph nodes | 18 | 18 | 11 | 29 | 0 | 7 | 95 | 28 | 26 | 42 | 11 | 12 | 26 | 5 | -21 | 0,506 | 0,845 | 0,696 |
| ≥ 10 involved lymph nodes | 8 | 7 | 5 | 55 | 0 | 0 | 62 | 48 | 44 | 20 | 28 | 31 | -13 | 21 | -19 | 0,807 | 0,528 | 0,794 |
| **Hormon receptor status** |  | | | | | | | | | | | | | | | | | |
| negative | 53 | 37 | 36 | 9 | 0 | 0 | 66 | 38 | 81 | 16 | 13 | 27 |  |  |  |  |  |  |
| positive | 121 | 113 | 84 | 12 | 0 | 0 | 69 | 36 | 45 | 13 | 7 | 15 | -42 | 41 | -95 | 0,517 | 0,287 | 0,257 |
| **Estrogen receptor status** |  | | | | | | | | | | | | | | | | | |
| negative | 62 | 47 | 42 | 9 | 0 | 0 | 66 | 47 | 70 | 14 | 16 | 24 | 4 | 58 | -38 | 0,938 | 0,052 | 0,581 |
| positive | 112 | 103 | 78 | 14 | 0 | 0 | 70 | 31 | 48 | 14 | 6 | 16 |  |  |  |  |  |  |
| **Progesterone receptor status** |  | | | | | | | | | | | | | | | | | |
| negative | 72 | 52 | 50 | 7 | 0 | 0 | 59 | 34 | 70 | 12 | 10 | 20 | -44 | -8 | -36 | 0,227 | 0,716 | 0,428 |
| positive | 102 | 98 | 70 | 14 | 0 | 0 | 75 | 38 | 48 | 15 | 8 | 18 |  |  |  |  |  |  |
| **Tumorsize** |  | | | | | | | | | | | | | | | | | |
| pT1a-c | 86 | 66 | 58 | 14 | 0 | 0 | 72 | 42 | 51 | 15 | 12 | 17 |  |  |  |  |  |  |
| pT2 | 81 | 77 | 56 | 8 | 0 | 0 | 66 | 32 | 63 | 15 | 7 | 23 | -11 | -15 | 18 | 0,644 | 0,303 | 0,542 |
| pT3 | 7 | 7 | 6 | 0 | 4 | 0 | 44 | 38 | 39 | 27 | 22 | 31 | -54 | -27 | 9 | 0,375 | 0,433 | 0,899 |
| **Histology** |  | | | | | | | | | | | | | | | | | |
| Invasive ductal carcinoma | 152 | 130 | 105 | 10 | 0 | 0 | 65 | 39 | 51 | 10 | 7 | 14 |  |  |  |  |  |  |
| Invasive lobular carcinoma | 12 | 10 | 7 | 18 | 0 | 47 | 117 | 34 | 61 | 79 | 19 | 34 | 66 | 6 | 23 | 0,148 | 0,840 | 0,712 |
| **Grading** |  | | | | | | | | | | | | | | | | | |
| G1 | 5 | 3 | 2 | 15 | 0 | 29 | 94 | 0 | 29 | 68 | 0 | 29 |  |  |  |  |  |  |
| G2 | 77 | 71 | 55 | 8 | 0 | 0 | 79 | 37 | 61 | 19 | 10 | 26 | -27 | 28 | 24 | 0,688 | 0,563 | 0,828 |
| G3 | 92 | 76 | 63 | 14 | 0 | 0 | 58 | 37 | 52 | 11 | 8 | 12 | -57 | 32 | 1 | 0,408 | 0,523 | 0,993 |
| **HER2/neu status** |  | | | | | | | | | | | | | | | | | |
| negative | 121 | 100 | 79 | 10 | 0 | 0 | 67 | 34 | 52 | 13 | 8 | 13 | -11 | -6 | -18 | 0,652 | 0,669 | 0,573 |
| positive | 52 | 49 | 38 | 13 | 0 | 0 | 74 | 42 | 67 | 17 | 9 | 32 |  |  |  |  |  |  |

(number [n], median [Q_0,50_], standard error [SE], regression coefficient [R])

Supplementary Table 8. Plasma VEGF-A165b in breast cancer patients.

|  | **VEGF-A165b (plasma)** | | | | | | | | | | | | | | | | | |
| --- | --- | --- | --- | --- | --- | --- | --- | --- | --- | --- | --- | --- | --- | --- | --- | --- | --- | --- |
|  | n | | | Q_0,50_ (pg/ml) | | | mean (pg/ml) | | | SE | | | R | | | p-value | | |
| Blood sample | 1 | 2 | 3 | 1 | 2 | 3 | 1 | 2 | 3 | 1 | 2 | 3 | 1 | 2 | 3 | 1 | 2 | 3 |
| **Nodal status** |  | | | | | | | | | | | | | | | | | |
| nodal-negative (N0) | 45 | 50 | 55 | 0 | 0 | 20 | 36 | 26 | 94 | 14 | 8 | 21 |  |  |  |  |  |  |
| 1-3 involved lymph nodes | 37 | 60 | 47 | 2 | 0 | 15 | 46 | 42 | 47 | 14 | 13 | 47 | 2 | 19 | 23 | 0,932 | 0,288 | 0,697 |
| 4-9 involved lymph nodes | 12 | 16 | 11 | 13 | 0 | 39 | 38 | 21 | 15 | 16 | 10 | 15 | -5 | 5 | -30 | 0,868 | 0,834 | 0,726 |
| ≥ 10 involved lymph nodes | 4 | 7 | 4 | 58 | 57 | 46 | 60 | 59 | 40 | 30 | 23 | 40 | 18 | 39 | -12 | 0,712 | 0,249 | 0,930 |
| **Hormon receptor status** |  | | | | | | | | | | | | | | | | | |
| negative | 33 | 33 | 34 | 0 | 0 | 46 | 42 | 35 | 121 | 18 | 11 | 32 |  |  |  |  |  |  |
| positive | 65 | 100 | 83 | 0 | 0 | 15 | 40 | 34 | 89 | 9 | 8 | 27 | 39 | 54 | 61 | 0,414 | 0,207 | 0,660 |
| **Estrogen receptor status** |  | | | | | | | | | | | | | | | | | |
| negative | 41 | 40 | 39 | 0 | 0 | 38 | 50 | 45 | 134 | 18 | 18 | 37 | 59 | 73 | 170 | 0,107 | 0,056 | 0,140 |
| positive | 57 | 93 | 78 | 0 | 0 | 15 | 34 | 30 | 81 | 7 | 6 | 26 |  |  |  |  |  |  |
| **Progesterone receptor status** |  | | | | | | | | | | | | | | | | | |
| negative | 45 | 46 | 49 | 0 | 0 | 18 | 37 | 33 | 84 | 14 | 9 | 23 | -14 | -4 | -60 | 0,653 | 0,868 | 0,395 |
| positive | 53 | 87 | 68 | 0 | 0 | 26 | 44 | 35 | 102 | 10 | 9 | 33 |  |  |  |  |  |  |
| **Tumorsize** |  | | | | | | | | | | | | | | | | | |
| pT1a-c | 48 | 60 | 60 | 0 | 0 | 16 | 42 | 39 | 97 | 14 | 13 | 25 |  |  |  |  |  |  |
| pT2 | 46 | 67 | 52 | 2 | 0 | 32 | 39 | 29 | 102 | 10 | 7 | 38 | -6 | -13 | 22 | 0,746 | 0,396 | 0,644 |
| pT3 | 4 | 6 | 5 | 21 | 37 | 10 | 49 | 50 | 73 | 36 | 23 | 44 | 1 | -9 | -75 | 0,984 | 0,808 | 0,565 |
| **Histology** |  | | | | | | | | | | | | | | | | | |
| Invasive ductal carcinoma | 85 | 113 | 101 | 0 | 0 | 16 | 44 | 37 | 93 | 10 | 8 | 24 |  |  |  |  |  |  |
| Invasive lobular carcinoma | 7 | 10 | 7 | 4 | 0 | 96 | 22 | 37 | 100 | 12 | 15 | 43 | -26 | 5 | 54 | 0,503 | 0,853 | 0,624 |
| **Grading** |  | | | | | | | | | | | | | | | | | |
| G1 | 3 | 3 | 2 | 0 | 0 | 67 | 26 | 0 | 67 | 26 | 0 | 67 |  |  |  |  |  |  |
| G2 | 43 | 61 | 55 | 4 | 0 | 13 | 50 | 39 | 122 | 16 | 12 | 42 | 26 | 25 | 12 | 0,645 | 0,613 | 0,948 |
| G3 | 52 | 69 | 60 | 0 | 0 | 38 | 34 | 32 | 78 | 9 | 7 | 14 | 1 | 24 | -44 | 0,984 | 0,633 | 0,807 |
| **HER2/neu status** |  | | | | | | | | | | | | | | | | | |
| Negative | 68 | 90 | 78 | 0 | 0 | 19 | 31 | 0 | 94 | 11 | 9 | 20 | 10 | -11 | -17 | 0,629 | 0,499 | 0,737 |
| Positive | 29 | 42 | 36 | 0 | 0 | 26 | 42 | 5 | 114 | 9 | 10 | 54 |  |  |  |  |  |  |

(number [n], median [Q_0,50_], standard error [SE], regression coefficient [R])
